# A verbal and social autopsy study to improve estimates of mortality determinants in Pakistan: study design and methods

**DOI:** 10.7189/jogh.16.05004

**Published:** 2026-09-11

**Authors:** Imran A Chauhadry, Sajid Bashir Soofi, Muhammad Atif Habib, Arjumand Rizvi, Ahmad Khan, Kanza Naqvi, Henry D Kalter, Robert E Black, Zulfiqar A Bhutta

**Affiliations:** 1 Centre of Excellence in Women and Child Health, Aga Khan University, Karachi, Pakistan; 2 Department of Paediatrics & Child Health, Aga Khan University, Karachi, Pakistan; 3 Institute for Global Health and Development, Aga Khan University, Karachi, Pakistan; 4 Centre for Global Child Health, The Hospital for Sick Children, Toronto, Ontario, Canada; 5 Institute for International Programs, Bloomberg School of Public Health, Johns Hopkins University, Baltimore, Maryland, USA

## Abstract

**Background:**

Reducing newborn, child, adolescent, and maternal mortality is a global priority, yet Pakistan continues to bear a high burden of preventable deaths. Routine civil registration does not capture causes or social determinants for most of these deaths. The verbal and social autopsy (VASA) methodology fills this gap by combining symptom-based identification of the cause of death with structured assessment of social and care-seeking factors. Here we describe the methodological framework of a national VASA study conducted using the Pakistan National Nutrition Survey 2018 sampling frame, designed to estimate cause-specific mortality and social determinants of stillbirths and neonatal, post-neonatal, adolescent, and maternal deaths

**Methods:**

We used the sampling frame of the Pakistan National Nutrition Survey 2018 and employed VASA techniques to identify biological causes and social determinants of stillbirths, neonatal, child, adolescent, and maternal deaths. By utilising adapted VASA tools and rigorous analytical methods, we sought to bridge the gap between health systems and mortality data by examining deaths that occurred in and outside of healthcare facilities. Our aim was to provide insights into preventable deaths to guide public health interventions and policymaking, such as integrating VASA into the national survey and building the capacity of community-level healthcare workers to conduct VASA.

**Conclusions:**

This study provides a vital contribution to improving health outcomes and reducing mortality in low- and middle-income countries by integrating sociocultural and systemic factors into mortality analysis.

**Keywords:**

verbal and social autopsy, cause of death, VASA methodology, Pakistan

Reducing mortality rates among newborns, children under five years of age, and women of reproductive age remains a pressing challenge in low- and middle-income countries (LMICs). Progress in reducing maternal and newborn deaths has stalled for the past eight years; Central and Southern Asia and sub-Saharan Africa regions currently bear the highest burden in this context, with 60% of global maternal deaths, stillbirths, and newborn deaths occurring in just 10 countries, while accounting for 51% of global live births [1]. These deaths are predominantly caused by preventable or treatable conditions and non-biological, *i.e.* social, economic, and environmental factors.

While the importance of recording vital events, such as births, deaths, and causes of death, through enhanced civil registration and vital statistics (CRVS) systems is well established [2,3], LMICs still face significant gaps in such efforts. Decades of underinvestment in these systems have left one in two deaths remaining unreported globally, with most of these unrecorded deaths occurring in LMICs. Despite efforts made to strengthen health information systems, identifying specific causes and determinants of mortality in resource-constrained settings remains challenging due to gaps in data availability, particularly for non-facility-based deaths; weak data recording mechanisms at facilities; and suboptimal CRVS systems. Understanding the causes and determinants of such deaths is critical for developing effective public health interventions, particularly in LMICs, where neonatal, under five years of age, and maternal mortality rates remain high. These data are likewise important for updating epidemiological estimates of mortality across age groups, which are often left unrecorded by health systems in these regions [4].

Pakistan has one of the highest mortality rates for neonates and children under five years of age globally, and one of the higher perinatal and maternal mortality rates among LMICs. According to the 2018 Pakistan Demographic and Health Survey (PDHS) [5], the country has a perinatal mortality rate of 57 per 1,000 pregnancies at gestation of seven or more months, a neonatal mortality rate of 42 per 1,000 live births, an infant mortality of 62 per 1,000 live births, and an under-five mortality rate of 74 per 1,000 live births [5]. The 2019 Pakistan Maternal Mortality Survey reports the maternal mortality among ever-married women aged 15–49 years to be 186 deaths per 100,000 live births [6]. Despite incremental improvements, these statistics reflect persistent gaps in health service coverage, quality of care, and accessibility – issues compounded by a paucity of reliable data on biological and social determinants of these deaths. While national surveys provide broad mortality estimates, they often lack granularity regarding specific causes of death and contributing social factors, limiting policymakers’ ability to design targeted interventions to reduce preventable mortality effectively. The misclassification of stillbirths, neonatal deaths, and other mortality events further undermines the accuracy of mortality data, resulting in an incomplete understanding of the health challenges faced by vulnerable populations.

Pakistan has primarily relied on large-scale household surveys for estimating mortality rates. National efforts such as the PDHS and the Pakistan Maternal Mortality Survey conducted in the 1990s and early 2000s provided critical insights into mortality trends, despite challenges with recall bias and sample limitations [7], revealing significant gaps in the official death registration system and prompting policymakers to consider complementary methods for capturing more accurate mortality data.

Meanwhile, district-level initiatives in Punjab and Sindh have worked towards modernising death registration by streamlining administrative processes and engaging local communities. Progress in these initiatives was slow and uneven due to infrastructural and bureaucratic hurdles, and efforts often fell short, resulting in fragmented and sometimes unreliable mortality statistics [8].

The verbal and social autopsy (VASA) methodology helps address these gaps by providing a structured framework to identify, determine, and analyse both the biological causes and social determinants of mortality. It combines verbal autopsy (VA) and social autopsy (SA) techniques to provide a deeper understanding of mortality determinants, making it more relevant in the context of LMICs, where barriers to accessing healthcare services, delays in seeking care, and inadequacies in health systems are often linked with biological causes of mortality. Evidence suggests that incorporating social determinants into mortality analyses improves the understanding of causes of death, enabling targeted public health interventions and policy responses [9]. The social determinants of mortality in Pakistan have remained largely unchanged over the years, and include factors such as low education, wealth inequalities, social taboos, and limited access to quality healthcare. However, these determinants have recently been exacerbated by economic downturns, high inflation, urbanisation, and rapid climate changes [10,11].

VA identifies the biological causes of death through caregiver-reported symptoms and physician coding or algorithmic analyses. In this approach, caregivers of the deceased are asked structured questions that focus on the signs and symptoms experienced by the deceased during their illness [12]. SA records social determinants beyond the medical causes of mortality, such as behavioural, sociocultural, and systemic factors that contributed to delays or failures in seeking appropriate care before a person’s death [13–15]. Such social determinants may not be evident when identifying biomedical causes alone; by integrating these approaches, this VASA study connects biological and social dimensions of mortality, offering a more nuanced perspective on preventable deaths.

The literature on VASA methodologies shows that a diversity of tools, combinations, and frameworks have been used and adapted to local contexts in various LMICs [16–21]. Similarly, the World Health Organization’s (WHO) VA tools, adapted for LMICs, have been widely used to estimate causes of death where vital registration systems are weak [9]. Meanwhile, SA frameworks have evolved to investigate delays in the continuum of care, including delays in recognising illness, seeking care, and receiving adequate treatment. Combining these approaches, the VASA methodology provides a holistic framework for understanding the multifactorial nature of mortality in LMICs and for guiding future strategies and policies.

## METHODS

We adapted the VASA methodology to the Pakistani context for this national study. We conducted the study using the sampling framework of the Pakistan National Nutrition Survey (PNNS) 2018 [22], which identified mortality cases across diverse demographic groups, including stillbirth (pregnancies terminating at ≥7 months of gestation), neonatal deaths (0–28 days), post-neonatal and child deaths (29 days to 59 months), older child and adolescent deaths (12–19 years), and maternal deaths (12–49 years). By employing this methodology, we aimed to contribute to a deeper understanding of mortality determinants and to inform policies and programmes designed to reduce preventable deaths across the life course.

### Objectives

We aimed to estimate the proportion of antepartum and intrapartum stillbirths and ascertain the cause-specific mortality distributions for neonatal, post-neonatal, child and adolescent, and maternal deaths. We also aimed to evaluate the behavioural and social factors influencing healthcare access to identify and understand the underlying determinants of mortality.

### Study design

We conducted the VASA study using the PNNS 2018 sampling frame, which included enumeration blocks from both urban and rural areas of four provinces (Sindh, Punjab, Baluchistan, and Khyber Pakhtunkhwa) and the administrative regions of Azad Jammu and Kashmir, Gilgit Baltistan, and Islamabad Capital Territory. The Pakistan Bureau of Statistics has divided the country into enumeration blocks comprising 200–250 houses on average. A total of 5,780 enumeration blocks were sampled from the national sampling frame for PNNS 2018 [22]. From these enumeration blocks, we used 4,475 blocks to conduct a census to identify the number of births, stillbirths and age-specific number of deaths, including neonatal deaths (0–28 days), post-neonatal deaths (29 days to 59 months), child and adolescent (5–19 years), and maternal deaths (12–49 years) during the three years prior to the survey (2015–2018). From the households where stillbirths and deaths were identified through the census, we randomly selected the required sample for conducting detailed VASA interviews.

The VASA study used a cross-sectional design with retrospective recall, examining deaths that occurred within the three years preceding the survey, capturing critical data on biological causes and social and behavioural factors. The VA and SA techniques ensured an analysis of the pathways leading to death, including health-seeking behaviours, social constraints, and systemic gaps in seeking and receiving healthcare. The target population included households with stillbirths or neonatal, post-neonatal, child, adolescent, and maternal deaths [23].

### Inclusion and exclusion criteria

We included respondents belonging to a household identified by the PNNS 2018 census as having experienced a recent stillbirth, neonatal, post-neonatal, child, adolescent, or maternal death. Respondents also needed to provide consent to participate in the interview and have information about the illness or the leading cause of death. They were also required to meet specific age criteria: for neonatal and post-neonatal and child deaths, the primary respondent was the mother (12–49 years); in case the mother was not present, the respondent had to be any caregiver aged 15 years and above. For adolescents and maternal deaths, the respondent had to be 15 years or older.

### Sample size

**Figure 1:**
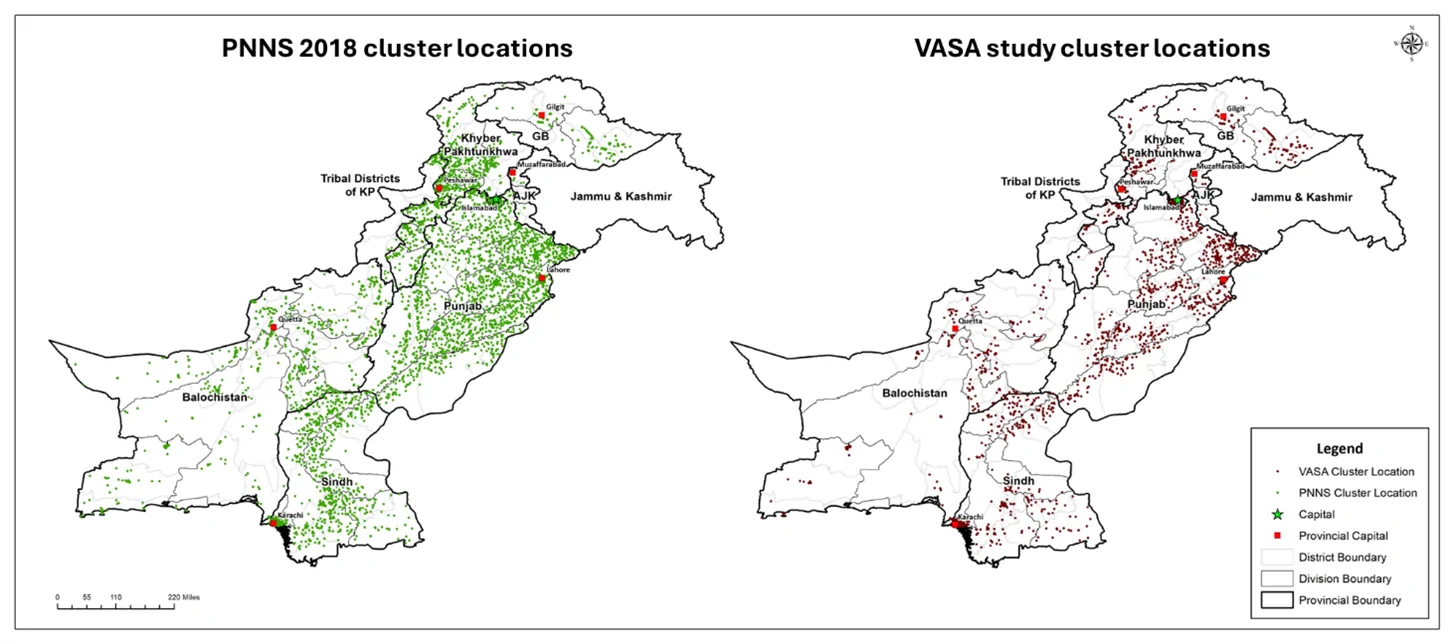
Geographic distribution of survey clusters included in the PNNS 2018 (**Panel A**) and VASA study (**Panel B**).

The VASA study was built on the PNNS 2018 [22], which covered 115,600 households across Pakistan. Using this extensive platform, we aimed to identify and understand the causes of death among neonates (0–28 days), post-neonates (29 days to 59 months), children and adolescents (12–19 years), and married women (12–49 years). We determined the sample size for the ascertainment of causes of death based on the precision of the estimates, calculating it for stillbirths and neonatal and post-neonatal deaths to achieve 3% precision for the most common cause of death in each age group. As true proportions were unknown, we assumed a 50% proportion to ensure we arrived at the largest sample size, which we then inflated for a design effect of 1.5 and a 15% non-response rate, resulting in a sample size of 1,884, rounded to 2,100 deaths in each category (stillbirths, neonatal deaths, and child and adolescent deaths). The overall VASA sample was then distributed to each province and region in proportion to population size (**Figure 1**, **Table 1**). Maternal and adolescent deaths were not independently powered; they were ascertained through the same household enumeration used for stillbirths and neonatal deaths, with achieved precision for these outcomes reported in the corresponding analysis papers. A computer-generated programme randomly selected the required number of households among households where deaths were identified.

**Table 1:** VASA sample size distribution across provinces and regions of Pakistan

Provinces/regions	Stillbirths	Neonatal deaths	Child deaths
Punjab	743	743	743
Sindh	344	344	344
Baluchistan	340	340	340
Khyber Pakhtunkhwa	295	295	295
Federally administered tribal areas	92	92	92
Gilgit Baltistan	126	126	126
Azad Jammu and Kashmir	160	160	160
**Total**	2100	2100	2100

### Study tools, their adaptation and translation

The VASA questionnaires used in this study were structured instruments consisting mostly of closed-ended questions. These questions were designed to be administered uniformly and without bias, ensuring objectivity and accurate information. The structured format helped systematically capture data on biological and social determinants of mortality. A similar approach to using structured questionnaires was observed in the Countrywide Mortality Surveillance for Action Mozambique study, where VASA tools were successfully employed. This facilitated community-based mortality surveillance through systematic data collection and structured questionnaires [24].

The VA component of this study questionnaire was adopted from the WHO 2016 VA Questionnaire, tailored to capture cause-specific mortality through a structured sequence of symptoms and event-based questions [25]. For stillbirths and neonatal deaths, the VA examines maternal health, delivery conditions, and early life care. For older children, adolescents, and adults, the VA identifies biological causes of death, such as pneumonia, diarrhoea, injuries, and maternal complications.

The SA component, adapted for the local context, was based on the Johns Hopkins Bloomberg School of Public Health/Institute for International Programs (JHU/IIP) SA Tool. It explores the behavioural and social determinants, including delays in seeking care, barriers to accessing healthcare, and the quality of services received [26]. Utilising the Pathway to Survival framework, the SA identifies points at which preventive or curative actions were lacking, contributing to mortality [14,15].

Experienced personnel translated the questionnaire from English into Urdu, Pakistan’s national language, while another group then independently back-translated it into English. Discrepancies identified in this process were reconciled through discussions between the translator and back-translator. Each corrected translation was then checked by a local anthropologist or other social scientist to ensure that key terms, such as signs or symptoms of illness, were correctly translated and that correct terms were used for local informal and formal healthcare providers. If necessary, the social scientist checked the translation of key local terms with formal and informal healthcare providers and child caregivers. The final tools were piloted in the field to refine them based on the feedback.

### Recruitment process

We identified households where pregnancies were terminated at seven or more months of gestation (likely stillbirths) or where a child under five years of age had passed away in the past three years through the PNNS census. We recorded deaths of children, adolescents, and mothers through interviews with the most appropriate respondents available during the survey visits. We included any household that experienced a stillbirth, one or more deaths in the past three years, with separate interviews conducted for each qualifying death.

Interviewers were carefully trained to select the most appropriate respondent in the household, as outlined in the recruitment scripts. Interviewers explained the criteria to respondents or other family members during household visits to ensure clarity.

During these visits, interviewers identified the primary caregiver of the stillborn, deceased child, adolescent, or married woman. Using recruitment scripts, they invited these caregivers to participate in the study. The interviewers followed different consent procedures depending on their age and marital status. Married individuals aged 12–17 and those 18 or older gave oral consent, while unmarried individuals aged 12–17 required parental permission in addition to their assent. After completing this process, interviewers formally invited the respondents to participate in the study.

### Consent process

We did not interview individuals who could not provide informed consent. While no formal assessment of ability was conducted, data collectors used their judgment to determine if potential respondents understood the consent process. For stillbirths and child deaths under 5 years, respondents had to be at least 12 years old, and for older children, adolescents, and maternal deaths, they had to be at least 18 years old. Data collectors identified the most knowledgeable potential respondent, read the informed consent (or assent and parental permission for minors) statement, addressed any questions to the respondent’s satisfaction, and proceeded with the interview only after consent or assent was provided.

### Data collection

The data collection involves a Computer-Assisted Personal Interview (CAPI) system to ensure accuracy and efficiency [27]. We developed an Android-compatible data collection application, which had built-in range, skip logic, and consistency checks to ensure data quality. Data was transmitted to the Aga Khan University (AKU) data centre in real time. Households with identified deaths were revisited, and the VASA interviews were conducted by trained field staff using handheld devices. Collaboration between the VASA and PNNS teams ensured that interviews were conducted promptly while respecting a culturally appropriate 40-day mourning period following a death. All households where one or more stillbirths or deaths occurred in the past three years were selected for VASA interviews, with interviews conducted for each qualifying death.

In each selected household, the VASA interviewer identified the most appropriate respondent for the interview, prioritising the mother of the deceased for stillbirths, neonatal deaths, and post-neonatal deaths and a knowledgeable family member aged 18 years or older for older children, adolescents, and maternal deaths. If the primary respondent was unavailable, an alternate respondent familiar with the deceased’s circumstances and who met the age requirements was sought. Recruitment scripts guided the interviewers in identifying respondents, ensuring consistency and clarity.

Upon arrival at the household, the interviewer explained the study’s purpose, identified the respondent, and sought oral consent for participation. Depending on the respondent’s age and marital status, the interviewer administered either the informed consent process or assent and parental permission. In cases where the respondent was unavailable or unable to complete the interview at the time of the visit, appointments were scheduled for follow-up visits.

VASA interviews were structured to follow a chronological narrative, allowing respondents to recount events leading to the death naturally and with minimal recall bias. The interviews were conducted using handheld computing devices to ensure accurate and efficient data capture. While most interviews were completed in a single visit, in rare cases, multiple visits were required to accommodate respondent availability or to include additional respondents necessary to complete the interview. Interviews typically lasted between an hour and a half. The CAPI application ensured that none of the required fields was left unanswered.

### Training

VASA interviewers were carefully selected and extensively trained to ensure high-quality data collection. Interviewers were adult men and women aged 18 years and above with at least a secondary school education and significant prior experience in household surveys. 

We ensured that the interviewers were aware of the sensitive nature of the information being gathered and the possible emotional impact of the interview on the respondents. The interviewers reflected this in their approach to the community and respondents. As such, training covered not only technical aspects but also relevant ethical issues, matters of sensitivity, and locally prescribed assistance to bereaved respondents

### Ethical considerations

The VASA study adhered to rigorous ethical standards to ensure participants’ dignity, safety, rights, and confidentiality. We obtained informed consent from eligible respondents aged 18 years or older, while respondents aged 12–17 years required parental permission and the respondent's assent. To maintain privacy and confidentiality, data were encrypted and securely stored, with protocols in place for data backup. We monitored data integrity through regular quality control procedures to ensure that data collection adhered to ethical and methodological standards. We obtained ethical approval from the Ethical Review Committee of the AKU and the National Bioethics Committee of the government of Pakistan.

### Data analysis

Interpretation of the verbal autopsy data was undertaken using multiple approaches to assign the underlying cause of death. These included the physician certified verbal autopsy (PCVA), in which trained physicians independently review VA records and assign causes of death based on the reported signs, symptoms, and circumstances of death, as well as computer-coded verbal autopsy (CCVA) methods, which were performed using one or more of InterVA-5, a probabilistic Bayesian model, expert algorithm verbal autopsy, tariff method, and InSilicoVA, a Bayesian statistical model that estimates both individual causes of death and population-level cause-specific mortality fractions [23,28–30]. Each approach has distinct strengths and limitations. The PCVA incorporates clinical judgment but is resource-intensive and subject to inter-physician variability, while the CCVA provide standardised, reproducible, and scalable cause-of-death assignment but rely on probabilistic relationships between reported symptoms and underlying causes of death. The agreement and differences between PCVA and CCVA methods were assessed to evaluate the consistency of cause-of-death assignment and to inform the interpretation of mortality patterns in the study population. Results from the different methods were examined to provide complementary estimates of cause-specific mortality and to assess the robustness of the findings across analytical approaches.

Two independent physicians trained in using the VA tool to ascertain the cause of death, but not engaged in the data collection, were tasked to assign the cause of death by using a standardised case definition and ICD-10 codes [31]. These physicians independently reviewed the completed verbal autopsy tool and the open history recorded at the time of the interview to assess the cause of death. To ensure objectivity, they were blinded to each other during the assessment process; where they disagreed, the case was referred to a third senior study physician for adjudication. In cases where a consensus could not be reached among the three physicians, or where available information was insufficient to categorise the cause of death, the case was labelled as 'undetermined'.

These physicians classified and coded biological causes of death by identifying direct and precipitating causes (such as pneumonia or birth asphyxia) and contributing factors that indirectly influenced the outcome. They also determined the single underlying cause of death that initiated the chain of events leading to the fatality. A coding scheme based on WHO recommendations and additional guidelines developed for the VASA study supported this process. To ensure the accuracy and consistency of results, the physicians received training to analyse VA data, utilising WHO materials and the VASA-specific guidelines.

We developed a customised web-based application to support physicians’ review for cause ascertainment. This application facilitated the automated assignment of VA cases to physicians in batches, where each case was assigned to two physicians for independent review. The physicians were also blinded by the assignment of cases. This web-based application also assigned discrepant cases to senior physicians.

In addition to VA, we analysed the SA data using the Pathway to Survival conceptual framework [14,15], identifying critical steps needed to restore health and examining where these failed. Originally designed to evaluate child deaths, the framework was adapted in this study to include maternal, neonatal, and adolescent deaths and stillbirths. The updated SA questionnaire explored recognition and care-seeking behaviours for maternal pregnancy and labour or delivery complications, normal newborn care practices, and preventive childcare. We used Cohen’s kappa to assess inter-rater reliability between the first and second reviewers.

The SA analysis quantified actions taken or missed at various points in the care pathway for stillbirths, neonates, children, adolescents, or mothers. It examined key decisions made during fatal illnesses, such as the first action taken for labour or delivery complications, the primary decision-maker for seeking care, and the reasons for delays or deviations from formal healthcare pathways. The focus was on delays in care-seeking, especially those caused by limited recognition of illness signs or reliance on alternative care options such as traditional healers. The analysis also explored how social and cultural factors, such as caregiver autonomy or perceptions of illness, influenced decisions to seek care.

By combining the VA and SA data, we provided insights into both the biological causes of mortality and its social determinants. The mortality rates for each target group estimated from the identified number of births and deaths correspond to the rates reported by the PDHS 2018 [5]. Detailed findings on mortality estimates from this VASA study will be published in separate papers. 

## DISCUSSION

The VASA study combined verbal and social autopsy methods to better understand the causes and contributing factors behind mortality in Pakistan. Using the PNNS 2018 sampling frame, we analysed a large, representative data set to examine deaths across different age groups. The findings provide crucial evidence to support the design of targeted public health interventions to reduce mortality rates.

By adapting the methodology to Pakistan’s context, we ensured ethical and culturally appropriate data collection. By combining VA and SA, we not only identified biological causes of death, but also examined the delays, social barriers, and healthcare gaps that contributed to these outcomes. This dual focus provided a better understanding of mortality by addressing immediate and underlying factors. The use of standardised tools, such as the WHO VA questionnaire [25] and JHU/IIP SA tool [26], also helped keep the study aligned with global best practices, ensuring that its findings could inform local and international health policies.

Similar cross-sectional community-based studies were conducted in other LMICs using the adapted VASA tool to ascertain the cause of death and identify social determinants [32]. India has incorporated VA into its Sample Registration System for mortality surveillance [33]. While efforts have also been made to integrate social determinants through community inquiries, Bangladesh’s maternal and perinatal death review system, for example, also integrates VASA [34], signifying the importance of such a technique in LMICs for cause of death assessment. Sub-Saharan African countries like Tanzania, Mozambique, and Ethiopia have also embraced VASA or similar methodologies within their health and demographic surveillance systems. These adaptations have highlighted the flexibility of VASA methods in accommodating diverse epidemiological and social environments. VASA methods have evolved worldwide and have been recognised as essential tools for capturing high-quality data, with importance placed on their adaptation to local contexts [15,35–37].

### Strengths and limitations

One significant strength of our study is its ability to identify deaths across various age groups and time frames. This allowed for detailed insights into recent mortality trends and the broader socio-behavioural factors influencing outcomes, such as delays in care-seeking and healthcare quality. These findings highlight the critical role that cultural norms and systemic challenges play in maternal and child health, recurring themes in similar global studies [12–17]. Using trained data collectors and CAPI was another strength, as it helped minimise errors and ensure consistent data collection.

However, this study has some limitations. Recall bias from respondents and the emotional nature of the interviews may have affected some responses and underreporting of events, particularly maternal deaths. Despite these limitations, structured interviewer training and ethical protocols helped reduce these risks and maintain data reliability.

### Public health and policy implications

Our results have important implications for public health policies and programmes. By identifying delays along the Pathway to Survival framework [14,15], the findings provide actionable insights into improving care-seeking behaviours and healthcare access.

By combining detailed cause-of-death information with socio-behavioural context, VASA enables health authorities to pinpoint gaps in service delivery and identify systemic barriers that lead to preventable deaths. For example, the integration of VASA in countries like India, Bangladesh, and several sub-Saharan African nations has revealed critical delays in care-seeking, deficiencies in emergency services, and cultural factors that hinder access to timely treatment. These insights can inform targeted interventions such as enhancing primary healthcare in underserved areas, improving emergency response protocols, and tailoring community outreach programs to address local barriers.

Similar VASA methodologies could be incorporated into civil registration and vital statistics systems and health and demographic surveillance systems to ensure continuous, high-quality mortality surveillance. Along with this, policies should be put in place to train healthcare workers in such methodologies, and the data collected through this system must be translated into actionable insights and responses [14,15].

## CONCLUSIONS

We provided an innovative approach to understanding mortality in Pakistan by addressing biological and social determinants using the PNNS 2018 platform, which enabled data capture across diverse demographic groups, enhancing the granularity and relevance of mortality analysis. We highlighted preventable deaths by leveraging adapted tools and robust analytical methods, offering actionable insights to address systemic gaps and sociocultural barriers in healthcare access. This framework underscores the importance of integrating social determinants into health research, setting a foundation for reducing preventable deaths and achieving sustainable health outcomes in LMICs. By building on this process, healthcare providers and policymakers can work towards significantly improving health outcomes in Pakistan and similar contexts worldwide.
